# District hospital experience of organ support requirements for H1N1-associated pneumonia

**DOI:** 10.1186/cc10660

**Published:** 2012-03-20

**Authors:** A Krige, S Chukkambotla

**Affiliations:** 1 East Lancashire Hospitals NHS Trust, Blackburn, UK

## Introduction

The objective of our study was to describe the disease pattern, outcomes and organ support required in treating H1N1-associated pneumonia in a single-centre, district hospital ICU.

## Methods

All of the patients with confirmed H1N1 infection admitted to our ICU during the months of December 2010 and January 2011 were studied. The outcome measures were incidence, severity and support for organ dysfunction, length of stay in ICU and mortality.

## Results

During the study period 27 patients were admitted. The mean age was 46.6 years (SD 13.6) with 20 (74%) patients being female, of whom two were pregnant. The mean APACHE scores were similar between survivors and nonsurvivors, 14.1 and 13.7 respectively. Twenty patients (74%) required invasive mechanical ventilation with median duration of 9 days (range 2 to 54 days). Advanced techniques like prone position ventilation and high-frequency oscillatory ventilation were required in 20% and 10% of these patients respectively. Two patients were referred for ECMO. Ventilator-associated pneumonia (VAP) ensued in 25% of invasively ventilated patients resulting in an increase in ventilator days (median) from 9 to 19 and ICU stay (median) from 15 to 23 days. Four (15%) required advanced cardiovascular support, 14 (52%) developed acute kidney injury (AKI) of which nine (33%) patients required renal replacement therapy. The ICU mortality was 11.1% and hospital mortality was 14.8%. The cohort who developed AKI had 21% mortality. The median ICU stay (range) was 15 days (2 to 68 days).

## Conclusion

H1N1 pneumonia was associated with significant morbidity and mortality requiring advanced multiorgan support in the majority of patients. Although the incidence of organ dysfunction in our cohort mirrored that found in the Swift study [1], in keeping with advances in management of H1N1-associated critical illness the mortality was lower in the current study.
